# Postoperative analgesia efficacy of erector spinae plane block in adult abdominal surgery: A systematic review and meta-analysis of randomized trials

**DOI:** 10.3389/fmed.2022.934866

**Published:** 2022-10-04

**Authors:** Yuzheng Gao, Lidan Liu, Yuning Cui, Jiaxin Zhang, Xiuying Wu

**Affiliations:** Department of Anesthesiology, Shengjing Hospital, China Medical University, Shenyang, China

**Keywords:** erector spinae plane block, abdominal surgery, nerve block, opioid consumption, anesthesia

## Abstract

**Objectives:**

Erector spinae plane block (ESPB) has been used for many thoracic and abdominal surgeries. However, evidence of its analgesic efficacy following abdominal surgery, compared with that of thoracic analgesia, is insufficient. Our study explored the analgesic effect of ESPB after abdominal surgery.

**Methods:**

We searched PubMed, Embase, Cochrane Central Register of Controlled Trials, and ClinicalTrials.gov. Primary outcomes were pain scores at 6, 12 and 24 h and 24-h opioid consumption. Secondary outcomes included time to first rescue analgesia, length of hospital stay, and incidence of postoperative nausea and vomiting (PONV). We calculated standardized mean differences (SMDs) with 95% confidence intervals (CIs) for primary outcomes and mean differences (MDs) and risk ratios (RRs) with 95% CIs for secondary outcomes.

**Results:**

We systematically included 1,502 cases in 24 trials. Compared with placebo, ESPB significantly reduced pain scores at 6 h (SMD −1.25; 95% CI −1.79 to −0.71), 12 h (SMD −0.85; 95% CI −1.33 to −0.37) and 24 h (SMD −0.84; 95% CI −1.30 to −0.37) and 24-h opioid consumption (SMD −0.62; 95% CI −1.19 to −0.06) post-surgery. ESPB prolonged the time to first rescue analgesia and decreased the incidence of PONV. Compared with transversus abdominal plane block (TAPB), ESPB significantly reduced pain scores at 6, 12, and 24 h and 24-h opioid consumption and prolonged the time to first rescue analgesia postsurgically. Furthermore, subgroup analysis showed that ESPB significantly reduced pain scores at various time points and opioid consumption within 24 h after laparoscopic cholecystectomy, percutaneous nephrolithotomy and bariatric surgery.

**Conclusion:**

Compared with placebo, ESPB improves the postoperative analgesic efficacy after abdominal surgery. Furthermore, our meta-analysis confirmed that ESPB provides more beneficial analgesic efficacy than TAPB.

**Systematic review registration:**

[https://www.crd.york.ac.uk/PROSPEROFILES/301491_STRATEGY_20220104.pdf], identifier [CRD42022301491].

## Introduction

Abdominal surgery is one of the most common surgical procedures clinically, and postoperative pain is a foreseeable problem. Although epidural analgesia yields good analgesic effects in major open abdominal surgery (1–4), its application is limited by the use of coagulants (5), which have unforeseeable effects on blood coagulation and compromise the safety of neuraxial techniques (6). In recent years, clinical guidelines have proven that nerve block has a better benefit/risk ratio (RR) than central neuraxial blocks and have recommended that nerve block should be performed to relieve pain after primary thoracoabdominal surgeries (7, 8). However, transversus abdominal plane block (TAPB) has several drawbacks as the nerve block is currently mainly used for abdominal surgery. For example, the needle tip may pierce the transversus abdominis (and peritoneum), injuring the internal organs and peritoneum while inducing TAPB (9).

Erector spinae plane block (ESPB) was first reported in 2016 by Forero et al. (10) and has gained much attention due to its safety and ease of application. In this technique, the local anesthetic (LA) is injected into the fascia between the erector spinae and the transverse process and diffuses in this fascia, which can block the nearby spinal nerve. ESPB not only affects the dorsal and ventral rami of spinal nerves and causes temporary loss of sensation in the corresponding body surface sensory areas innervated by them but also affects the rami communicants that transmit sympathetic fibers. It has been proven that ESPB provides both somatic and visceral sensory blocks of the abdomen (10, 11), which makes it an ideal nerve block for abdominal surgery.

There has been an increasing amount of new evidence regarding ESPB’s effectiveness in preventing pain during abdominal surgery. However, thus far, most meta-analyses have focused mainly on validating the effects of ESPB in thoracic or breast surgery and comparing them with thoracic paravertebral blocks, there is a lack of studies exploring their effectiveness in abdominal surgery or comparing them with other trunk blocks such as TAPB. The current meta-analyses (12–15) only included a small number of studies involving abdominal surgery and Daghmouri et al. (16) only researched the effect of ESPB in laparoscopic cholecystectomy (LC).

Therefore, our systematic review and meta-analysis aimed to determine the analgesic effect of ESPB after abdominal surgery. We identified randomized controlled trials (RCTs) comparing ESPB with either placebo or TAPB.

## Methods

This systematic review and meta-analysis were performed and reported in compliance with the Preferred Reporting Items for Systematic Reviews and Meta-Analyses (17). Our meta-analysis was registered prospectively with PROSPERO (CRD42022301491).

### Search strategy

Literature searches were performed using PubMed, Embase, the Cochrane Central Register of Controlled Trials, and the ClinicalTrials.gov register from 2016 until 24 September 2021 for English RCTs meeting the listed inclusion criteria, as ESPB is a new regional nerve block first introduced in 2016. The search used the MeSH keywords “Paraspinal Muscles,” “Cardiac Surgical Procedures,” “Nerve Block,” and “Anesthesia, Local.” The detailed search strategy is provided in Supplementary Appendix A.

### Study selection criteria

The two authors (GZ and LL) independently screened the search results and included trials that met the following criteria: (i) adult patients (age ≥18 years) treated with abdominal surgery, including LC, percutaneous nephrolithotomy (PCNL) and bariatric surgery (BS), etc., under general anesthesia; (ii) interventions: treatment with a single-injection ESPB with LA before or after surgery; and (iii) controls: placebo (no block and sham block) and TAPB. (iv) One or more of the following outcomes were assessed: postoperative pain scores at 6, 12 and 24 h, 24-h postoperative cumulative opioid consumption (mg), the incidence of postoperative nausea and vomiting (PONV) within 24 h postoperatively; length of hospital stay (days); time to first rescue analgesia (hours). (v) Only studies published in English were included. (vi) Only studies that were RCTs were included.

In our meta-analysis, trials were excluded that met the following criteria: (i) studies which did not provide available data (ii) studies which were withdrawn;

### Data extraction

Two investigators (GZ and LL) independently reviewed the full manuscripts of eligible studies and conducted data extraction using a standardized form. Extracted data included the author names, publication year; sample size; type of surgery; unilateral or bilateral; comparator(s); LA type, concentration, and volume; timing of block (before or after surgery); guidance of ESPB (ultrasound-guided or fluoroscopy-guided); postoperative outcomes including postoperative pain scores, 24-h cumulative opioid consumption, time to first rescue analgesia, length of hospital stay, and incidence of PONV. Any discrepancies regarding the extraction of data were resolved by an additional investigator (XW). When the pain score data were absent, they were replaced by the pain score data during movement, and if they were still absent, the pain score data were replaced by the pain score data at rest. If patients in the intervention and control groups received the same nerve block, this nerve block was not considered in this analysis.

To facilitate meta-analysis, medians, interquartile ranges (IQRs), and range values were approximated into standardized mean differences (SMDs) and mean differences (MDs) with their corresponding SDs. If data values were represented in a graphical format, numerical data were extracted from graphs by Web Plot Digitizer (18). The risk of bias assessment was independently assessed by two investigators, with any disagreements judged by a third investigator (XW), according to the Cochrane Collaboration tool for assessing the risk of bias (19). Studies were assessed on randomization, allocation concealment, participant and personnel blinding, outcome assessment blinding, incomplete data and selective reporting; each category of the study was assigned “low risk,” “high risk,” or “unclear risk.”

### Outcome measurement

Our primary outcomes were postoperative pain scores at 6, 12, and 24 h, as well as 24-h cumulative opioid consumption. Pain scores were measured by a visual or numerical scale (0–10 scale, where 0 = no pain and 10 = worst pain imaginable). Any visual analog scale (VAS) scores reported on a 0–100 scale were converted to a 0–10 scale for analysis. All reported perioperative opioid consumption was converted to intravenous morphine equivalents (20). Our secondary outcomes were the time to first rescue analgesia measured by hours after surgery, days of hospital stay after surgery, and incidence of PONV within 24 h postoperatively.

### Data analysis

All meta-analyses were conducted using Review Manager V5.4.1. (Cochrane Collaboration, Copenhagen) and Stata 16.0 software. For continuous data of primary outcomes, including postoperative pain scores and 24-h cumulative opioid consumption, SMDs with a 95% confidence interval (95% CI) were calculated, but for continuous data of secondary outcomes, including length of hospital stay and time to first rescue analgesia, MDs with 95% CIs were calculated. Dichotomous data are presented by using RRs with 95% CIs.

If *I*^2^ > 50%, differences would be regard as significant (21). The random-effects model was used for all outcomes, and forest plots were used to represent and evaluate treatment effects. Subgroups were created to explore and resolve potential heterogeneity within the intervention and control groups based on the type of surgery, the timing of the block (before surgery or after surgery), and ESPB techniques (bilateral or unilateral). Subgroup analysis was performed if the number of studies included was not less than two. For outcomes with the data of ten or more studies, Egger’s regression (DerSimonian–Laird approach) was used to assess potential publication bias of the small-study effect. Moreover, a sensitivity analysis was performed by removing each study in turn to evaluate the stability of pooled estimate. Sensitivity analyses were performed for those studies with a high degree of heterogeneity (*I*^2^ ≥ 50% or *P* < 0.1). Finally, pooled analyses were visualized with forest plots and tables and *P* < 0.05 was considered statistically significant.

## Results

**FIGURE 1:**
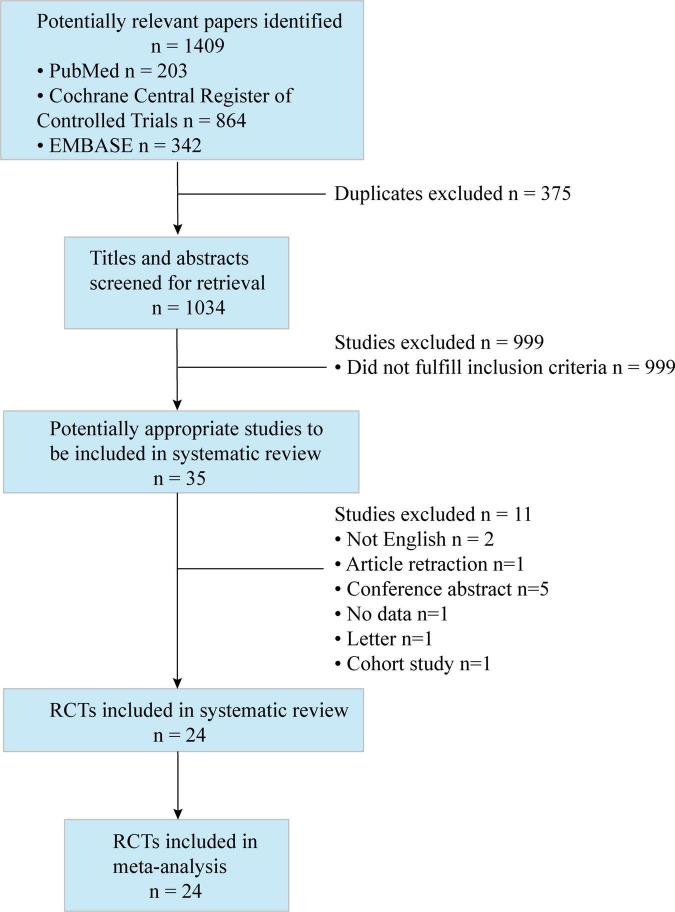
Preferred reporting items for systematic reviews and meta-analyses (PRISMA) flow diagram. The diagram shows the process and the reason for excluding studies.

**FIGURE 2:**
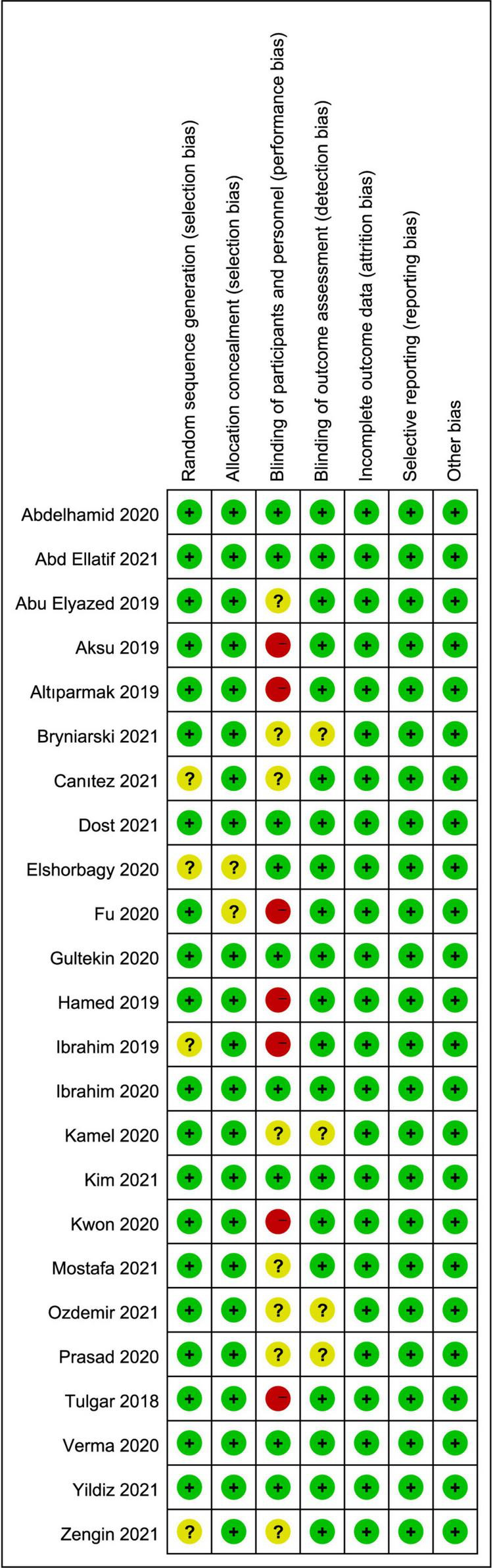
Risk of bias summary: review authors’ judgments about each risk of bias item for each included study. Green circle, low risk; red circle, high risk; yellow circle, unclear risk of bias.

A total of 1,409 studies were identified by our search criteria, and 375 duplicates were removed. Of the 1,034 remaining studies screened, 24 studies (22–45) were included in this review (Figure 1), with a total of 1,502 patients (701 who received ESPB, 801 who did not). The risk of bias assessment is summarized in Figure 2. The main sources of bias from the included studies were the lack of a description of participant and personnel blinding.

The characteristics of the included studies are summarized in Table 1. There were twenty RCTs (22–25, 27–32, 34, 36–39, 41–45) that compared ESPB with placebo, six studies (23, 26, 33, 35, 39, 40) that compared ESPB with TAPB. Abdominal surgeries were performed under general anesthesia in all studies: in nine studies (25, 26, 28, 33, 37, 40, 42–44) for LC, in four studies (27, 31, 34, 41) for PCNL, in three studies (23, 38, 45) for BS, in two studies (30, 36) for laparoscopic hepatectomy (LH), in two studies (32, 35) for total abdominal hysterectomy, in one study (22, 24) for hernia repair, in one study (22) for open nephrectomy, in one study (29) for open radical prostatectomy and in one study (39) for emergency laparotomy. Moreover, in the majority (20 of the 24) of the studies (22–27, 29–34, 36–40, 42, 43, 45), ESPB was performed before the surgery. Bilateral ESPB was conducted in 18 of the 24 studies (23, 24, 26, 28–30, 32, 33, 35–40, 42–45), while unilateral ESPB was used in the remaining studies (6 of the 24) (22, 25, 27, 31, 34, 41).

**TABLE 1:** Overview of included studies’ characteristics: ESPB vs. placebo and ESPB vs. TAPB.

Study	Sample	Type of surgery	ESPB group		Control group		Block timing	Guide	Outcome
						
			Intervention	Local analgesia drug	Control	Local analgesia drug			
Abdelhamid et al. (23)	66	Bariatric surgery	Bilateral ESPB (*n* = 22)	30 ml 0.25% bupivacaine (each side)	Bilateral STAPB (*n* = 22) No block (*n* = 22)	30 ml 0.25% bupivacaine (each side)	Before surgery	Ultrasound-guided	Pain scores; opioid consumption within 24 postoperative hours; time to first rescue analgesia; incidence of PONV
Abd Ellatif and Abdelnaby (22)	75	Open nephrectomy	Unilateral ESPB (*n* = 25)	0.3–0.4 ml/kg 0.25% bupivacaine with a maximum volume of 30 ml	No block (*n* = 25)	0.3–0.4 ml/kg 0.25% bupivacaine with a maximum volume of 30 ml	Before surgery	Ultrasound-guided	Opioid consumption within 24 postoperative hours; the first time to rescue analgesia; length of hospital stay
Abu Elyazed et al. (24)	60	Open epigastric hernia repair	Bilateral ESPB (*n* = 30)	20 ml 0.25% bupivacaine (each side)	Sham block (*n* = 30)	/	Before surgery	Ultrasound-guided	Pain scores; opioid consumption within 24 postoperative hours; time to first rescue analgesia; incidence of PONV;
Aksu et al. (25)	46	Laparoscopic cholecystectomy	Unilateral ESPB (*n* = 23)	20 ml 0.25% bupivacaine	No block (*n* = 23)	/	Before surgery	Ultrasound-guided	Pain scores; opioid consumption within 24 postoperative hours; incidence of PONV
Altıparmak et al. (26)	68	Laparoscopic cholecystectomy	Bilateral ESPB (*n* = 34)	20 ml 0.375% bupivacaine (each side)	OSTAPB (*n* = 34)	20 ml of 0.375% bupivacaine (each side)	Before surgery	Ultrasound-guided	Pain scores; opioid consumption within 24 postoperative hours; incidence of PONV
Bryniarski et al. (27)	68	Percutaneous nephrolithotomy	Unilateral ESPB (*n* = 34)	20 ml 0.5% bupivacaine	No block (*n* = 34)	/	Before surgery	Ultrasound-guided	Pain scores; opioid consumption within 24 postoperative hours; incidence of PONV
Canıtez et al. (28)	82	Laparoscopic cholecystectomy	Bilateral ESPB (*n* = 41)	20 ml consisting of 7.5 ml 0.5% bupivacaine + 2.5 ml 2% lidocaine + 10 ml 0.9% saline (each side)	No block (*n* = 41)	/	After surgery	Ultrasound-guided	Pain scores; opioid consumption within 24 postoperative hours; incidence of PONV
Dost et al. (29)	50	Open radical prostatectomy	Bilateral ESPB (*n* = 25)	10 ml 1% lidocaine + 10 ml 0.5% bupivacaine (each side)	Sham block (*n* = 25)	/	Before surgery	Ultrasound-guided	Pain scores; opioid consumption within 24 postoperative hours
NCT03989570 (39)	93	Emergency laparotomies	Bilateral ESPB + sham TAPB (*n* = 31)	ESPB with 40 ml 0.25% bupivacaine/TAPB with 40 ml 0.9% saline	Bilateral TAPB/sham ESPB (*n* = 31) No block (*n* = 31)	TAPB with 40 ml 0.25% bupivacaine/ESPB with 40 ml 0.9% saline	Before surgery	Ultrasound-guided	Opioid consumption within 24 postoperative hours; time to the first rescue analgesia
Fu et al. (30)	60	Laparoscopic hepatectomy	Bilateral ESPB (*n* = 30)	20 ml 0.5% ropivacaine (each side)	No block (*n* = 30)	/	Before surgery	Ultrasound-guided	Pain scores; length of hospital stay
Gultekin et al. (31)	60	Percutaneous nephrolithotomy	Unilateral ESPB (*n* = 30)	20 ml 0.5% bupivacaine	No block (*n* = 30)	/	Before surgery	Ultrasound-guided	Pain scores; opioid consumption within 24 postoperative hours; length of hospital stay; time to the first rescue analgesia
Hamed et al. (32)	60	Total abdominal hysterectomy	Bilateral ESPB (*n* = 30)	20 ml 0.5% bupivacaine (each side)	Sham block (*n* = 30)	/	Before surgery	Ultrasound-guided	Pain scores; opioid consumption within 24 postoperative hours; length of hospital stay
Ibrahim and Elnabtity (34)	50	Percutaneous nephrolithotomy	Unilateral ESPB (*n* = 25)	30 ml 0.25% bupivacaine	Sham block (*n* = 25)	/	Before surgery	Ultrasound-guided	Pain scores; opioid consumption within 24 postoperative hours; time to the first rescue analgesia; incidence of PONV
Ibrahim (33)	63	Laparoscopic cholecystectomy	Bilateral ESPB (*n* = 21)	20 ml 0.25% bupivacaine hydrochloride (each side)	OSTAP (*n* = 21)	20 ml 0.25% bupivacaine hydrochloride (each side)/ 40 ml 0.25% bupivacaine + sham block	Before surgery	Ultrasound-guided	Opioid consumption within 24 postoperative hours; time to the first rescue analgesia; incidence of PONV
Kamel et al. (35)	48	Total abdominal hysterectomy	Bilateral ESPB (*n* = 24)	20 ml bupivacaine 0.375% + 5 μg/ml adrenaline (each side)	Bilateral TAPB (*n* = 24)	20 ml of bupivacaine 0.375% + 5 μg/ml adrenaline (each side)	After surgery	Ultrasound-guided	Pain scores; opioid consumption within 24 postoperative hours; time to the first rescue analgesia; incidence of PONV
Kim et al. (36)	70	Laparoscopic hepatectomy	Bilateral ESPB (*n* = 35)	40 ml 0.5% ropivacaine	No block (*n* = 35)	/	Before surgery	Ultrasound-guided	Pain scores; opioid consumption within 24 postoperative hours; time to the first rescue analgesia; incidence of PONV
Kwon et al. (37)	53	Laparoscopic cholecystectomy	Bilateral ESPB (*n* = 26)	ESPB with 20 ml 0.20% ropivacaine (each side)	No block (*n* = 27)	15 ml 0.20% ropivacaine (each side)	Before surgery	Ultrasound-guided	Pain scores; opioid consumption within 24 postoperative hours; incidence of PONV
Mostafa et al. (38)	60	Bariatric surgery	Bilateral ESPB (*n* = 30)	20 ml 0.25% bupivacaine (each side)	Sham block (*n* = 30)	/	Before surgery	Ultrasound-guided	Pain scores; opioid consumption within 24 postoperative hours; time to the first rescue analgesia; incidence of PONV
Ozdemir et al. (40)	64	Laparoscopic cholecystectomy	Bilateral ESPB (*n* = 32)	10 ml 0.25% bupivacaine + 10 ml 2% prilocaine (each side)	Bilateral STAPB (*n* = 32)	10 ml 0.25% bupivacaine + 10 ml 2% prilocaine (each side)	Before surgery	Ultrasound-guided	Pain scores; opioid consumption within 24 postoperative hours; length of hospital stay
Prasad et al. (41)	61	Percutaneous nephrolithotomy	Unilateral ESPB (*n* = 31)	20 ml 0.375% ropivacaine	No block (*n* = 30)	/	After surgery	Fluoroscopy-guided	Pain scores; opioid consumption within 24 postoperative hours; time to the first rescue analgesia; incidence of PONV
Tulgar et al. (42)	30	Laparoscopic cholecystectomy	Bilateral ESPB (*n* = 15)	20 ml 0.375% bupivacaine (each side)	No block (*n* = 15)	/	Before surgery	Ultrasound-guided	Opioid consumption within 24 postoperative hours
Verma et al. (43)	84	Laparoscopic cholecystectomy	Bilateral ESPB (*n* = 42)	20 ml 0.375% ropivacaine (each side)	Sham block (*n* = 42)	/	Before surgery	Ultrasound-guided	Pain scores
Yildiz et al. (44)	68	Laparoscopic cholecystectomy	Bilateral ESPB (*n* = 34)	10 ml 0.5% bupivacaine + 5 ml 2% lidocaine + 5 ml isotonic saline (each side)	No block (*n* = 34)	/	After surgery	Ultrasound-guided	Pain scores; incidence of PONV
Zengin et al. (45)	63	Bariatric surgery	Bilateral ESPB (*n* = 31)	20 ml 0.5% bupivacaine + 5 ml 0.2% lidocaine (each side)	No block (*n* = 32)	/	Before surgery	Ultrasound-guided	Pain scores

PONV, postoperative of nausea and vomiting; ESPB, erector spinae plane block; STAPB, subcostal transversus abdominis plane block; OSTAPB, oblique subcostal transversus abdominis plane block; TAPB, transversus abdominis plane block; PONV, postoperative nausea and vomiting.

### Postoperative pain scores

**FIGURE 3:**
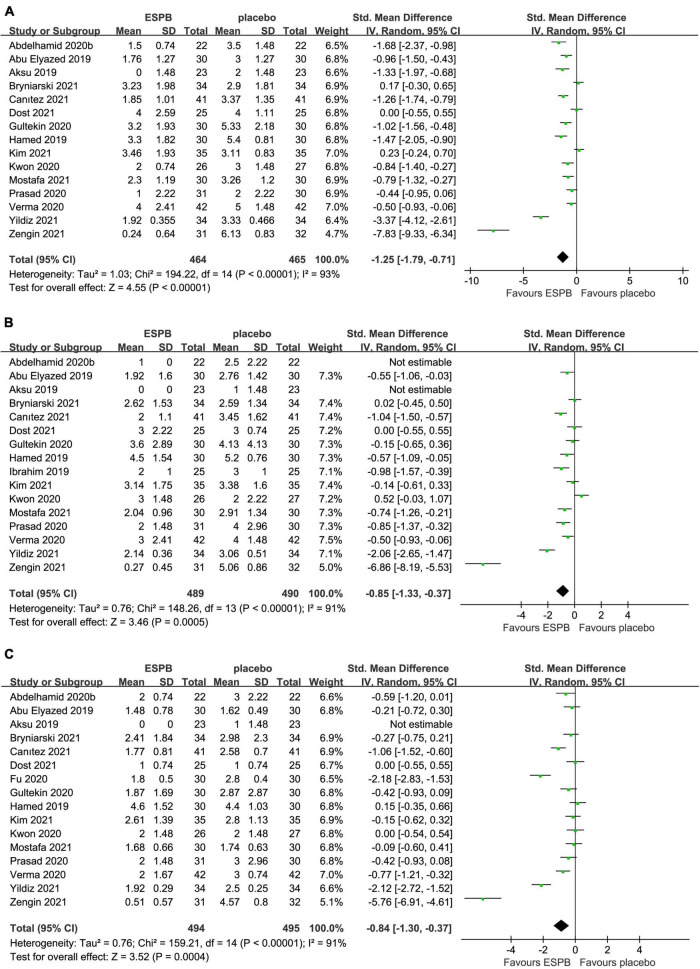
Forest plot of pain scores for the ESPB vs. placebo in the first 24 h after surgery. **(A)** Pain scores at 6 h after surgery. **(B)** Pain scores at 12 h after surgery. **(C)** Pain scores at 24 h after surgery.

**FIGURE 4:**
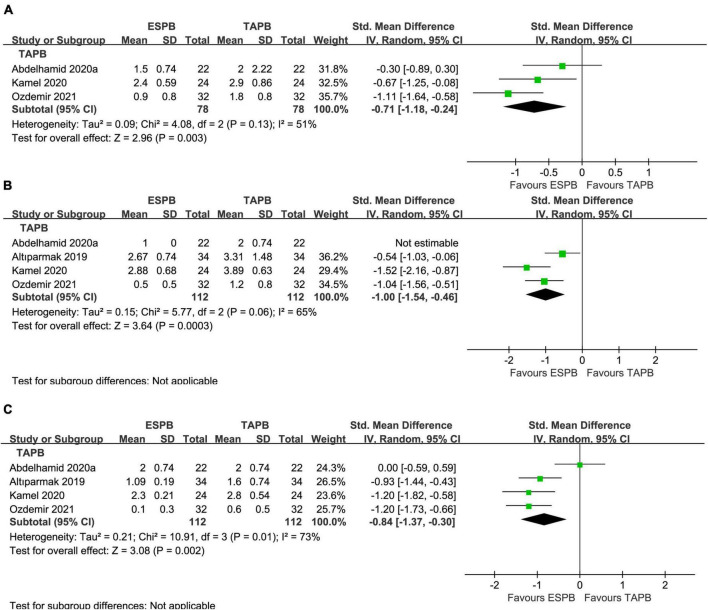
Forest plot of pain scores for the ESPB vs. TAPB in the first 24 h after surgery. **(A)** Pain scores at 6 h after surgery. **(B)** Pain scores at 12 h after surgery. **(C)** Pain scores at 24 h after surgery.

Compared with the placebo group, there was a significant reduction in postoperative pain scores in the ESPB group at various time points (Table 2): fifteen studies (23–25, 27–29, 31, 32, 36–38, 41, 43–46) reported significantly lower pain scores at 6 h (−1.25 cm; 95% CI −1.79 to −0.71; *P* < 0.00001; *I*^2^ = 93%) (Figure 3A). However, 16 studies (23–25, 27–29, 31, 32, 34, 36–38, 41, 43–45) reported significantly lower pain scores at 12 h (−0.85 cm; 95% CI −1.33 to −0.37; *P* = 0.0005; *I*^2^ = 91%) (Figure 3B) and 24 h (−0.84 cm; 95% CI −1.30 to −0.37; *P* = 0.0004; *I*^2^ = 91%) (Figure 3C). In our meta-analysis, compared with TAPB, ESPB significantly reduced pain scores at time points after abdominal surgery (Table 2): three trials (23, 35, 40) reported significantly lower pain scores at 6 h (−0.71 cm; 95% CI −1.18 to −0.24; *P* = 0.003; *I*^2^ = 51%) (Figure 4A) after abdominal surgery and four studies (23, 26, 35, 40) revealed significantly lower postoperative pain scores at 12 h (−1.00 cm; 95% CI −1.54 to −0.46 *P* = 0.0003; *I*^2^ = 65%) (Figure 4B) and 24 h (−0.84 cm; 95% CI −1.37 to −0.30; *P* = 0.002; *I*^2^ = 73%) (Figure 4C). Moreover, we conducted subgroup analyses to determine the postoperative analgesia conferred by ESPB compared with placebo in different types of surgery. The subgroup analysis of primary outcomes was performed as follows.

**TABLE 2:** Outcomes data for comparison of ESPB group versus placebo/TAPB group.

Comparison	Outcome	Participants	Trials	Relative effect (95% CI)	*I*^2^ (%)	*P*-values
ESPB vs. placebo	6-h pain scores	929	15	SMD −1.25 (−1.79, −0.71)	93	*P* < 0.00001
ESPB vs. placebo	12-h pain scores	979	16	SMD −0.85 (−1.33, −0.37)	91	*P* = 0.0005
ESPB vs. placebo	24-h pain scores	989	16	SMD −0.84 (−1.30, −0.37)	91	*P* = 0.0004
ESPB vs. placebo	24-h opioids	906	16	SMD −1.44 (−2.01, −0.87)	93	*P* < 0.00001
ESPB vs. placebo	Length of hospital stay	230	4	MD −0.31 (−0.69, 0.07)	81	*P* = 0.11
ESPB vs. placebo	Time to first rescue analgesia	494	9	MD 6.97 (4.92, 9.02)	100	*P* < 0.00001
ESPB vs. placebo	Incidence of PONV	662	11	RR 0.67 (0.46, 0.97)	20	*P* = 0.04
ESPB vs. TAPB	6-h pain scores	156	3	SMD −0.71 (−1.18, −0.24)	51	*P* = 0.003
ESPB vs. TAPB	12-h pain scores	224	4	SMD −1.00 (−1.54, −0.46)	65	*P* = 0.0003
ESPB vs. TAPB	24-h pain scores	224	4	SMD −0.84 (−1.37, −0.30)	73	*P* = 0.002
ESPB vs. TAPB	24-h opioids	308	6	SMD −1.85 (−2.54, −1.15)	81	*P* < 0.00001
ESPB vs. TAPB	Length of hospital stay	64	1	MD −0.13 (−0.18, −0.08)	/	*P* < 0.00001
ESPB vs. TAPB	Time to first rescue analgesia	240	5	MD 5.57 (0.03, 11.11)	99	*P* = 0.05
ESPB vs. TAPB	Incidence of PONV	182	4	RR 0.68 (0.26, 1.77)	0	*P* = 0.43

Outcomes data for comparison of ESPB group vs. placebo/TAPB group. ESPB, erector spinae plane block; TAPB, transversus abdominal plane block; PONV, postoperative nausea and vomiting; CI, confidence interval; MD, mean difference; SMD, standardized mean difference; RR, risk ratio.

In the subgroup analysis of LC, five trials (25, 28, 37, 43, 44) reported that, compared with placebo, ESPB significantly reduced pain scores at 6 h (−1.42 cm; 95% CI −2.23 to −0.60; *P* = 0.0006; *I*^2^ = 91%) and 24 h (−0.98 cm; 95% CI −1.74 to −0.21; *P* = 0.01; *I*^2^ = 89%) (Table 3). Interestingly, five trials (25, 28, 37, 43, 44) showed that no significant difference was detected in postoperative pain scores at 12 h (−0.62 cm; 95% CI −1.39 to 0.15; *P* = 0.11; *I*^2^ = 90%) between the groups (Table 3).

**TABLE 3:** Subgroup analysis of type of surgery.

Subgroup	Outcome	Trials	Participants	Relative effect (95% CI)	*I*^2^ (%)	*P*-values
ESPB vs. placebo for LC	6-h pain scores	5	333	SMD −1.42 (−2.23, −0.60)	91	*P* = 0.0006
ESPB vs. placebo for LC	12-h pain scores	5	333	SMD −0.62 (−1.39, 0.15)	90	*P* = 0.11
ESPB vs. placebo for LC	24-h pain scores	5	333	SMD −0.98 (−1.74, −0.21)	89	*P* = 0.01
ESPB vs. placebo for LC	24-h opioids	4	211	SMD −1.19 (−1.81, −0.56)	76	*P* = 0.0002
ESPB vs. placebo for PCNL	6-h pain scores	3	189	SMD −0.42 (−1.10, 0.25)	81	*P* = 0.22
ESPB vs. placebo for PCNL	12-h pain scores	4	239	SMD −0.49 (−0.97, −0.02)	70	*P* = 0.04
ESPB vs. placebo for PCNL	24-h pain scores	3	189	SMD −0.44 (−0.73, −0.15)	0	*P* = 0.003
ESPB vs. placebo for PCNL	24-h opioids	4	239	SMD −0.62 (−1.19, −0.06)	71	*P* = 0.03
ESPB vs. placebo for BS	6-h pain scores	3	167	SMD −3.22 (−5.95, −0.48)	97	*P* = 0.02
ESPB vs. placebo for BS	12-h pain scores	3	167	SMD −3.77 (−9.77, 2.23)	99	*P* = 0.22
ESPB vs. placebo for BS	24-h pain scores	3	167	SMD −2.08 (−4.59, 0.42)	97	*P* = 0.10
ESPB vs. placebo for BS	24-h opioids	2	104	SMD −2.57 (−3.10, −2.04)	0	*P* < 0.00001
ESPB vs. placebo for LH	24-h pain scores	2	130	SMD −1.59 (−4.46, 1.27)	98	*P* = 0.28
ESPB vs. placebo for LH	24-h opioids	1	70	SMD −0.13 (−0.60, 0.34)	/	*P* = 0.59

ESPB, erector spinae plane block; LC, laparoscopic cholecystectomy; PCNL, percutaneous nephrolithotomy; BS, bariatric surgery; LH, laparoscopic hepatectomy; CI, confidence interval; SMD, standardized mean difference.

In the subgroup analysis of PCNL, three studies (27, 31, 41) reported that, compared with placebo, ESPB provided comparable pain scores at 6 h (−0.42 cm; 95% CI −1.10 to 0.25; *P* = 0.22; *I*^2^ = 81%) and significantly lower postoperative pain scores at 24 h (−0.44 cm; 95% CI −0.73 to −0.15; *P* = 0.003; *I*^2^ = 0%) (Table 3). Meanwhile, four studies (27, 31, 34, 41) reported a significant reduction in postoperative pain scores at 12 h (−0.49 cm; 95% CI −0.97 to −0.02; *P* = 0.04; *I*^2^ = 70%) (Table 3) in the ESPB group after PCNL, compared with placebo.

In the subgroup analysis of BS, three trials (23, 38, 45) revealed that, compared with placebo, ESPB significantly reduced pain scores at 6 h (−3.22 cm; 95% CI −5.95 to −0.48; *P* = 0.02; *I*^2^ = 97%) (Table 3). However, no significant difference was found in postoperative pain scores at 12 h (−3.77 cm; 95% CI −9.77 to 2.23; *P* = 0.22; *I*^2^ = 99%) and 24 h (−2.08 cm; 95% CI −4.59 to 0.42; *P* = 0.10; *I*^2^ = 97%) after BS between the groups (Table 3).

In the subgroup analysis of LH, two studies (30, 36) found no significant difference in postoperative pain scores at 24 h (−1.59 cm; 95% CI −4.46 to 1.27; *P* = 0.28; *I*^2^ = 98%) between ESPB and placebo groups (Table 3). However, subgroup analysis was not performed due to the limited number of studies involving pain scores at 6 and 12 h after LH.

### Postoperative 24-h cumulative opioid consumption

**FIGURE 5:**
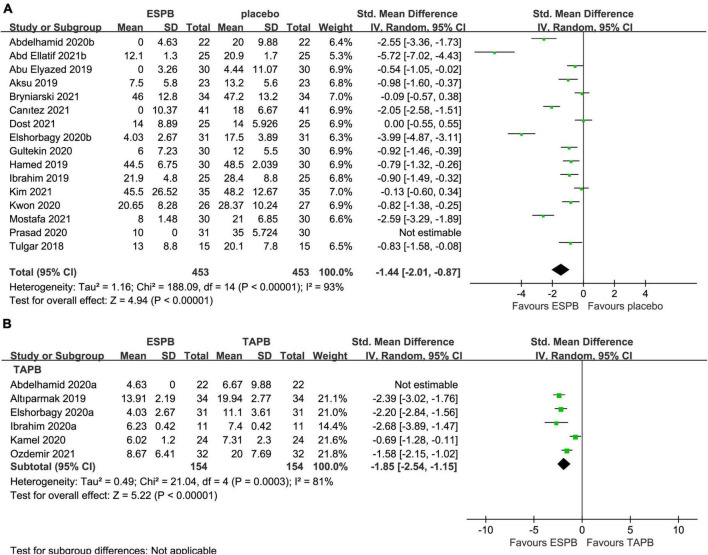
Forest plot for the comparison of intravenous morphine equivalents (mg) in the first 24 h after surgery. **(A)** Twenty-four hours cumulative opioid consumption for the ESPB vs. placebo studies. **(B)** Twenty-four hours cumulative opioid consumption for the ESPB vs. TAPB studies.

The 24-h cumulative opioid consumption after abdominal surgery was investigated in 16 studies (22–25, 27–29, 31, 32, 34, 36–39, 41, 42), with a significant reduction (−1.44 mg; 95% CI −2.01 to −0.87; *P* < 0.00001; *I*^2^ = 93%) in the ESPB group compared with the placebo group (Table 2 and Figure 5A).

The 24-h cumulative opioid consumption after abdominal surgery was investigated by six studies (23, 26, 33, 35, 39, 40), with a significant reduction in opioid intake (−1.85 mg; 95% CI −2.54 to −1.15; *I*^2^ = 81%; *P* < 0.00001) in the ESPB group compared with TAPB group (Table 2 and Figure 5B).

In the subgroup analysis of LC, four studies (25, 28, 37, 42) reported that, compared with placebo, ESPB significantly reduced the 24-h cumulative opioid consumption (−1.19 mg; 95% CI −1.81 to −0.56; *P* = 0.0002; *I*^2^ = 76%) after LC (Table 3).

In the subgroup analysis of PCNL, compared with placebo group, four studies (27, 31, 34, 41) reported that ESPB significantly reduced 24-h cumulative opioid consumption (−0.62 mg; 95% CI −1.19 to −0.06; *P* = 0.03; *I*^2^ = 71%) (Table 3).

In the subgroup analysis of BS, two studies (23, 38) revealed that, compared with placebo group, ESPB significantly reduced 24-h cumulative opioid consumption (−2.57 mg; 95% CI −3.10 to −2.04; *P* < 0.00001; *I*^2^ = 0%) (Table 3). However, due to the limitation of the number of studies involving 24-h cumulative opioid consumption after LH, subgroup analysis was not performed (Table 3).

### Secondary outcome measures

#### Time to first rescue analgesia

The time to first rescue analgesia after abdominal surgery was reported in nine trials (22–24, 31, 34, 36, 38, 39, 41), and compared with the placebo group, ESPB significantly prolonged the time to first rescue analgesia (6.97 h; 95% CI 4.92 to 9.02; *P* < 0.0001; *I*^2^ = 100%) (Table 2). Five studies (23, 33, 35, 39, 40) including 240 patients undergoing abdominal surgery reported that, compared with the TAPB group, ESPB significantly extended the time to first rescue analgesia (5.57 h; 95% CI 0.03 to 11.11; *P* = 0.05; *I*^2^ = 99%) (Table 2).

#### Length of hospital stay

Four trials (22, 30–32) compared the length of hospital stay of 230 patients undergoing abdominal surgery between the ESPB group and placebo group. However, the length of hospital stay was not significantly different (−0.31 days; 95% CI −0.69 to 0.07; *P* = 0.11; *I*^2^ = 81%) between the groups (Table 2). In one study by Ozdemir et al., which compared ESPB with TAPB for LC, there was a significantly shorter hospital stay in the ESPB group (40) (Table 2).

#### Incidence of postoperative nausea and vomiting

**FIGURE 6:**
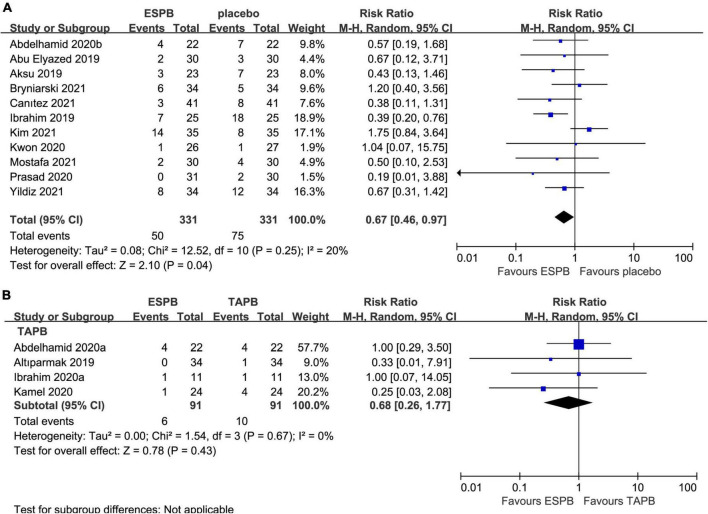
Forest plot for the comparison of the incidence of postoperative PONV. **(A)** Postoperative incidence of PONV for the ESPB vs. placebo studies. **(B)** Postoperative incidence of PONV for the ESPB vs. TAPB studies.

Eleven trials (23–25, 27, 28, 34, 36–38, 41, 44) reported the impact of ESPB on the incidence of PONV in 662 patients undergoing abdominal surgery. ESPB significantly reduced the incidence of PONV (RR 0.67; 95% CI 0.46 to 0.97; *P* = 0.04; *I*^2^ = 20%) compared with that in the placebo group (Table 2 and Figure 6A). In addition, four studies (23, 26, 33, 35) analyzed the incidence of PONV in patients receiving ESPB vs. TAPB. However, there was no significant difference in the incidence of PONV (RR 0.68; 95% CI 0.26 to 1.77; *P* = 0.43; *I*^2^ = 0%) between the groups (Table 2 and Figure 6B).

### Subgroup analysis of block techniques and timing of block

Subgroup analyses of block techniques (unilateral or bilateral ESPB) and the timing of block (before or after surgery) are presented in Table 4. Our meta-analysis revealed that performing ESPB after surgery significantly prolonged the time to first request for analgesia after abdominal surgery compared with that in the before-surgery subgroup (*P* = 0.002, DerSimonian–Laird approach). However, for other outcomes, the block technique and the timing of the block showed no statistical subgroup differences (*P* > 0.05).

**TABLE 4:** Subgroup analysis of timing of block (before/after surgery) and ESPB techniques (unilateral/bilateral).

Comparison	Outcome	Subgroup	Participants	Trials	Relative effect (95% CI)	*I*^2^ (%)	*P*-values	*P* for interaction
ESPB vs. placebo	6-h pain scores	Before surgery	718	12	SMD −1.15 (−1.73, −0.56)	92	*P* = 0.0001	0.474
ESPB vs. placebo	6-h pain scores	After surgery	211	3	SMD −1.66 (−3.12, −0.21)	95	*P* = 0.02	
ESPB vs. placebo	12-h pain scores	Before surgery	768	13	SMD −0.73 (−1.27, −0.18)	91	*P* = 0.010	0.297
ESPB vs. placebo	12-h pain scores	After surgery	211	3	SMD −1.30 (−1.98, −0.61)	80	*P* = 0.0002	
ESPB vs. placebo	24-h pain scores	Before surgery	778	13	SMD −0.75 (−1.28, −0.22)	91	*P* = 0.006	0.461
ESPB vs. placebo	24-h pain scores	After surgery	211	3	SMD −1.19 (−2.09, −0.29)	89	*P* = 0.010	
ESPB vs. placebo	24-h opioids	Before surgery	763	14	SMD −1.39 (−1.99, −0.80)	92	*P* < 0.00001	0.58
ESPB vs. placebo	24-h opioids	After surgery	143	2	SMD −2.05 (−2.58, −1.51)	93	*P* < 0.00001	
ESPB vs. placebo	Time to first rescue analgesia	Before surgery	456	8	MD 5.90 (4.04, 7.77)	100	*P* < 0.00001	0.002
ESPB vs. placebo	Time to first rescue analgesia	After surgery	38	1	MD 14.46 (13.78, 15.14)	100	*P* < 0.00001	
ESPB vs. placebo	Incidence of PONV	Before surgery	211	8	RR 0.72 (0.44, 1.19)	0	*P* = 0.20	0.46
ESPB vs. placebo	Incidence of PONV	After surgery	451	3	RR 0.54 (0.29, 1.03)	36	*P* = 0.06	
ESPB vs. placebo	6-h pain scores	Bilateral	694	11	SMD −1.51 (−2.23, −0.80)	94	*P* < 0.0001	0.184
ESPB vs. placebo	6-h pain scores	Unilateral	235	4	SMD −0.63 (−1.28, 0.01)	83	*P* = 0.06	
ESPB vs. placebo	12-h pain scores	Bilateral	694	11	SMD −1.04 (−1.70, −0.38)	93	*P* = 0.002	0.348
ESPB vs. placebo	12-h pain scores	Unilateral	285	5	SMD −0.47 (−0.96, 0.02)	71	*P* = 0.06	
ESPB vs. placebo	24-h pain scores	Bilateral	754	12	SMD −0.98 (−1.57, −0.39)	93	*P* = 0.001	0.33
ESPB vs. placebo	24-h pain scores	Unilateral	235	4	SMD −0.37 (−0.65, −0.08)	0	*P* = 0.01	
ESPB vs. placebo	24-h opioids	Bilateral	617	11	SMD −1.35 (−1.98, −0.72)	92	*P* < 0.0001	0.626
ESPB vs. placebo	24-h opioids	Unilateral	289	5	SMD −1.76 (−3.21, −0.32)	95	*P* = 0.02	
ESPB vs. placebo	Length of hospital stay	Bilateral	120	2	MD −1.18 (−3.52, 1.16)	90	*P* = 0.32	0.357
ESPB vs. placebo	Length of hospital stay	Unilateral	110	2	MD −0.22 (−0.76, 0.32)	61	*P* = 0.43	
ESPB vs. placebo	Time to first rescue analgesia	Bilateral	296	5	MD 8.79 (0.82, 16.76)	99	*P* = 0.03	0.305
ESPB vs. placebo	Time to first rescue analgesia	Unilateral	198	4	MD 5.01 (0.47, 9.55)	100	*P* = 0.03	
ESPB vs. placebo	Incidence of PONV	Bilateral	437	7	RR 0.80 (0.51, 1.25)	12	*P* = 0.33	0.231
ESPB vs. placebo	Incidence of PONV	Unilateral	225	4	RR 0.51 (0.28, 0.92)	14	*P* = 0.02	

ESPB, erector spinae plane block; PONV, postoperative nausea and vomiting; MD, mean difference; SMD, standardized mean difference; RR, risk ratio.

### Publication bias and sensitivity analysis

Egger’s test showed that the *P*-value for postoperative pain scores at 6, 12, and 24 h and for 24-h cumulative opioid consumption was less than 0.0001, which was less than 0.05 (Table 5). Therefore, some publication bias existed in the primary outcome and may have influenced the final result, but publication bias did not exist for the incidence of PONV.

**TABLE 5:** Egger’s test for outcomes.

Outcomes	Egger’s test *P*-value
Pain scores at 6 h	0.0000
Pain scores at 12 h	0.0000
Pain scores at 24 h	0.0000
24-h cumulative opioids consumption	0.0000
Incidence of PONV	0.5110

PONV, postoperative of nausea and vomiting.

According to the sensitivity analysis, most of overall outcomes did not change after the exclusion of a single study except postoperative pain scores at 6 h and time to first rescue analgesia between ESPB and TAPB (Supplementary Figures 2, 4).

## Discussion

This systematic review and meta-analysis showed the postoperative analgesic efficacy of ESPB in adults undergoing abdominal surgery under general anesthesia. When compared with placebo (e.g., no block and sham block), ESPB provided better postoperative analgesia at various time points and reduced opioid consumption within 24 h after surgery. Furthermore, ESPB was associated with a longer time to first rescue analgesia and a lower incidence of PONV postoperatively after abdominal surgery. However, it was not beneficial in shortening the length of hospital stay.

Compared with TAPB, ESPB also provided significantly lower pain scores at the various time points and lower opioid consumption within 24 h after surgery. Meanwhile, ESPB significantly prolonged the time to first rescue analgesia after abdominal surgery. However, we found no significant differences in the incidence of PONV between the groups.

Moreover, we tried to perform a subgroup analysis to explore the effect of ESPB on different types of surgery. Our meta-analysis showed that ESPB seems to be most beneficial in terms of reduction not only in pain scores but also in opioid consumption for patients undergoing LC, PCNL, and BS. Based on our meta-analysis, the best indication for performing ESPB for postoperative analgesia is LC (e.g., reduced postoperative pain at 6 and 24 h and 24-h cumulative opioid consumption) and PCNL (e.g., reduced pain at 12 and 24 h and 24-h cumulative opioid consumption). Similarly, ESPB could be recommended for BS (e.g., reduced pain at 6 h and 24-h postoperative cumulative opioid consumption). However, due to a limited number of studies, there is no effective recommendation for the effect of ESPB in reducing pain or opioid consumption in other types of surgery, such as LH, hernia repair, open nephrectomy, open radical prostatectomy or emergency laparotomy.

Due to the high heterogeneity of the outcomes, we also tried to perform subgroup analyses of the timing of block (before/after the surgery) and ESPB technique (unilateral/bilateral). The time to first rescue analgesia was significantly prolonged by ESPB in both the before-surgery and after-surgery subgroups, and the effect on the after-surgery subgroup was significantly more powerful than that in the before-surgery subgroup. However, the heterogeneity was still high (*I*^2^ = 100%) for both subgroups, and the number of studies for the after-surgery subgroup was limited (only one). Consequently, the results need to be confirmed by more research.

A cadaver study (10) reported the use of ESPB to inject LA into the fascia between the erector spinae and the transverse process; the LA was able to pass through the fascia to infiltrate and paralyze the spinal nerves. ESPB can act on dorsal and ventral branches of the spinal nerves and rami communicants that transmit sympathetic fibers. Due to the erector spinae and erector spinae plane extending down to the lumbar spine, ESPB can provide analgesia for abdominal surgery if the injection is performed at the lower levels of the thoracic spine. Recently, few cadaveric and radiological studies have described the LA diffusion range of ESPB. The results showed that ESPB seemed to work by spreading the LA to the epidural and paravertebral space. In this way, ESPB would be able to implement somatic and visceral analgesic effects such as epidural anesthesia (47–50). Moreover, the transverse process of the spine can now act as a puncture needle support point and anatomic landmark on ultrasound, which means ESPB is easy to perform (22, 26, 51).

A previous meta-analysis (16) that included 5 RCTs with 250 patients undergoing LC concluded that, compared with placebo, ESPB significantly decreased postoperative pain scores and 24-h cumulative opioid consumption as well as significantly prolonged the time to first rescue analgesia. In our analysis, we obtained similar results. We believe these findings suggest that ESPB plays an important role in postoperative analgesia for LC. Moreover, similar to our meta-analysis, a few previous meta-analyses (12–15) demonstrated that the ESPB group had significantly lower pain scores, lower 24-h cumulative opioid consumption, longer time to first rescue analgesia and lower incidence of PONV among patients undergoing surgery. However, these meta-analyses analyzed various surgeries, and as they only contained a small number of trials of abdominal surgery, they could not demonstrate the analgesic effect of ESPB in abdominal surgery. Our meta-analysis included 24 RCTs and performed a meta-analysis to compare ESPB with placebo or TAPB for postoperative analgesia in abdominal surgery patients based on a larger sample size. Moreover, the quality of trials in these meta-analyses should also be considered, as two of them (12, 15) included trials (52) that have been retracted.

This meta-analysis showed the beneficial effect and ease of application of postoperative analgesia compared with placebo and our sensitivity analysis also showed strong ability of the pooled analysis (Supplementary Figures 1, 3–5). ESPB has been applied in various kinds of surgeries, including lumbar spine surgery (53), LC (16), breast cancer surgery (54), and other thoracic and abdominal surgeries, and no side effects or complications related to this block have been reported. Our present meta-analysis provides novel evidence that ESPB is an effective nerve block for analgesia after abdominal surgeries.

In comparison with that of TAPB, the injection point of ESPB is remote from the peritoneum and abdominal wall and poses a lower risk of abdominal organ damage and peritoneal breach (9, 10). While ESPB provides somatic and visceral sensory block of the abdomen (10, 11), TAPB only supplies analgesia to the anterolateral abdominal wall (55). Therefore, ESPB may provide more effective analgesia after abdominal surgery. Due to the combination of its efficacy and lower risk of complications, ESPB has been regarded as an alternative to TAPB for postoperative analgesia in certain surgical operations. In addition, our review revealed that, compared with TAPB, ESPB is associated with a longer time to first rescue analgesia and a comparable incidence of PONV after abdominal surgery. However, perhaps due to the influence of different surgical types, different postoperative analgesia regimens and differences in clinician habits, time to first rescue analgesia can vary. For example, Ozdemir et al. (40) reported a longer time to first rescue analgesia in the TAPB group, while other studies report longer time in the ESPB group. It was worth mentioning that sensitivity analysis of postoperative pain scores at 6 h and time to first rescue analgesia found that the outcomes were not stable (Supplementary Figures 2, 4). Moreover, since not many studies have reported on these two outcomes, subgroup analysis was not performed to explore the effect of timing of block (before/after the surgery) and ESPB technique, so the veracity of both outcomes deserve further research. Even so, our meta-analysis still provides new evidence that ESPB may be a promising alternative to TAPB after abdominal surgery. However, the differences in analgesic effects and other postoperative anesthetic outcomes of both nerve blocks still require direct comparison in future large-volume and well-designed RCTs.

However, some limitations of our meta-analysis should be mentioned. First, the main drawback of our meta-analysis is that high heterogeneity was observed between studies. The sources of high heterogeneity also included differences in the types and doses of LAs, differences in multimodal analgesia, performer differences and patient differences (age, sex, etc.), etc. Second, Egger’s tests of primary outcomes revealed a high risk of small-study effects, which also reduced the reliability of our meta-analysis. Third, our meta-analysis only included studies involving abdominal surgeries instead of all kinds of surgical procedures. Therefore, the effect of postoperative analgesia may be exaggerated due to selection bias. Fourth, our meta-analysis only focused on the comparison of the ESPB group with the placebo or TAPB group. We did not compare the postoperative analgesic effect of ESPB with that of other postoperative analgesic methods (such as intrathecal morphine, quadratus lumborum block, or local infiltration). Therefore, more studies in this area are needed in the future. Fifth, the sample sizes of the studies included in this review were all relatively small. The largest sample size of the experimental group was only 42 patients. In the future, large-volume studies are needed in this area. Finally, relatively few studies have focused on the same surgery. Subgroup analyses by type of surgery were only applied to LC, PCNL, BS, and LH, with 6, 4, 3, and 2 RCTs, respectively.

## Conclusion

In summary, ESPB is a novel, beneficial nerve block for adult patients undergoing abdominal surgery. Moreover, our meta-analysis confirms that ESPB provides more beneficial postoperative analgesic efficacy than TAPB. Therefore, our research recommends ESPB as a supplement to the multimodal analgesic regimen for abdominal surgery and a valid alternative to TAPB. Future, large-volume, well-designed RCTs with extensive follow-up are needed to confirm and update the findings in this area.

## Data availability statement

The original contributions presented in this study are included in the article/Supplementary material, further inquiries can be directed to the corresponding author.

## Author contributions

YG and XW were responsible for the conception and design of the study, and drafting and revising the manuscript. YG, LL, and XW conducted the literature retrieval, screening, and quality evaluation. YG, YC, and JZ analyzed the data and explained the results. All authors contributed to the article and approved the submitted version.
