# Supplementary material for: Postoperative analgesia efficacy of erector spinae plane block in adult abdominal surgery: A systematic review and meta-analysis of randomized trials
Source: Front Med (Lausanne). 2022 Oct 4;9:934866. doi: 10.3389/fmed.2022.934866 (PMC9578553; doi:10.3389/fmed.2022.934866)
Supplement: Supplementary file 1 [file Data_Sheet_1.DOCX]

Supplementary Material

**Appendix A**

*PubMed Search Strategy*

#1 erector spinae plane or ESP block*

#2(erector or paraspinal or thoracic neuropathic pain) and block*

#3 erector spinae and (anesth*or anaesth* or analg*)

#4 paraspinal and ((regional or local) and (anesth* or anaesth* or analg*))

#5 Paraspinal Muscles[mesh] or Cardiac Surgical Procedures[mesh]

#6 Nerve Block[mesh] or Anesthesia, Local[mesh]

#7 #5 and #6

#8 #1 or #2 or #3 or #4 or #7

#9("Randomized Controlled Trial" [Publication Type] OR "Controlled Clinical Trial" [Publication Type] OR "Clinical Trials as Topic"[Mesh:NoExp] OR randomized[Title/Abstract] OR placebo [Title/Abstract] OR randomly[Title/Abstract] OR trial[Title/Abstract]) NOT ("Animals"[Mesh] NOT "Humans"[Mesh])

#10 #8 and #9

#11("2016"[Date - Create] : "3000"[Date - Create])

#12 #10 and#11

Retriever by :Yuzheng Gao Retrival date:9/24/2021

*Cochrane Central Register of Controlled Trials*

#1(erector spinae plane OR ESP block*):ti,ab,kw.

#2((erector OR paraspinal OR thoracic neuropathic pain) AND block*):ti,ab,kw.

#3(erector spinae AND (anaesth*OR analg*OR anesth*)):ti,ab,kw.

#4(paraspinal AND ((regional OR local) AND (anaesth* OR analg* OR anesth*))):ti,ab,kw.

#5MeSH descriptor: [Paraspinal Muscles] explode all trees

#6MeSH descriptor: [Cardiac Surgical Procedures] explode all trees

#7MeSH descriptor: [Nerve Block] explode all trees

#8MeSH descriptor: [Anesthesia, Local] explode all trees

#9#5OR#6

#10#7OR#8

#11#9AND#10

#12#1OR#2OR#3OR#4OR#11

#12 with Publication Year from 2016 to 2021, in Trials

*EMBASE Search Stratrgy*

#1'erector spinae plane':ab,ti OR 'esp block*':ab,ti.

#2(erector:ab,ti OR paraspinal:ab,ti OR 'thoracic neuropathic pain':ab,ti) AND block*:ab,ti.

#3'erector spinae':ab,ti AND (an?esth*:ab,ti OR analg*:ab,ti).

#4paraspinal:ab,ti AND (regional:ab,ti OR local:ab,ti) AND (an*esth*:ab,ti OR analg*:ab,ti).

#5'paraspinal muscle'/exp

#6'heart surgery'/exp

#7'nerve block'/exp

#8'local anesthesia'/exp

#9#5OR#6

#10#7OR#8

#11#9AND#10

#12#1OR#2OR#3OR#4OR#11

#13'crossover procedure':de OR 'double-blind procedure':de OR 'randomized controlled trial':de OR 'single-blind procedure':de OR random*:de,ab,ti OR factorial*:de,ab,ti OR crossover*:de,ab,ti OR ((cross NEXT/1 over*):de,ab,ti) OR placebo*:de,ab,ti OR ((doubl* NEAR/1 blind*):de,ab,ti) OR ((singl* NEAR/1 blind*):de,ab,ti) OR assign*:de,ab,ti OR allocat*:de,ab,ti OR volunteer*:de,ab,ti.

#14#12AND#13

#15#12 AND #13 AND [2016-2021]/py
