# Supplementary material for: Postoperative analgesia efficacy of erector spinae plane block in adult abdominal surgery: A systematic review and meta-analysis of randomized trials
Source: Front Med (Lausanne). 2022 Oct 4;9:934866. doi: 10.3389/fmed.2022.934866 (PMC9578553; doi:10.3389/fmed.2022.934866)
Supplement: Supplementary file 2 [file Data_Sheet_2.DOCX]

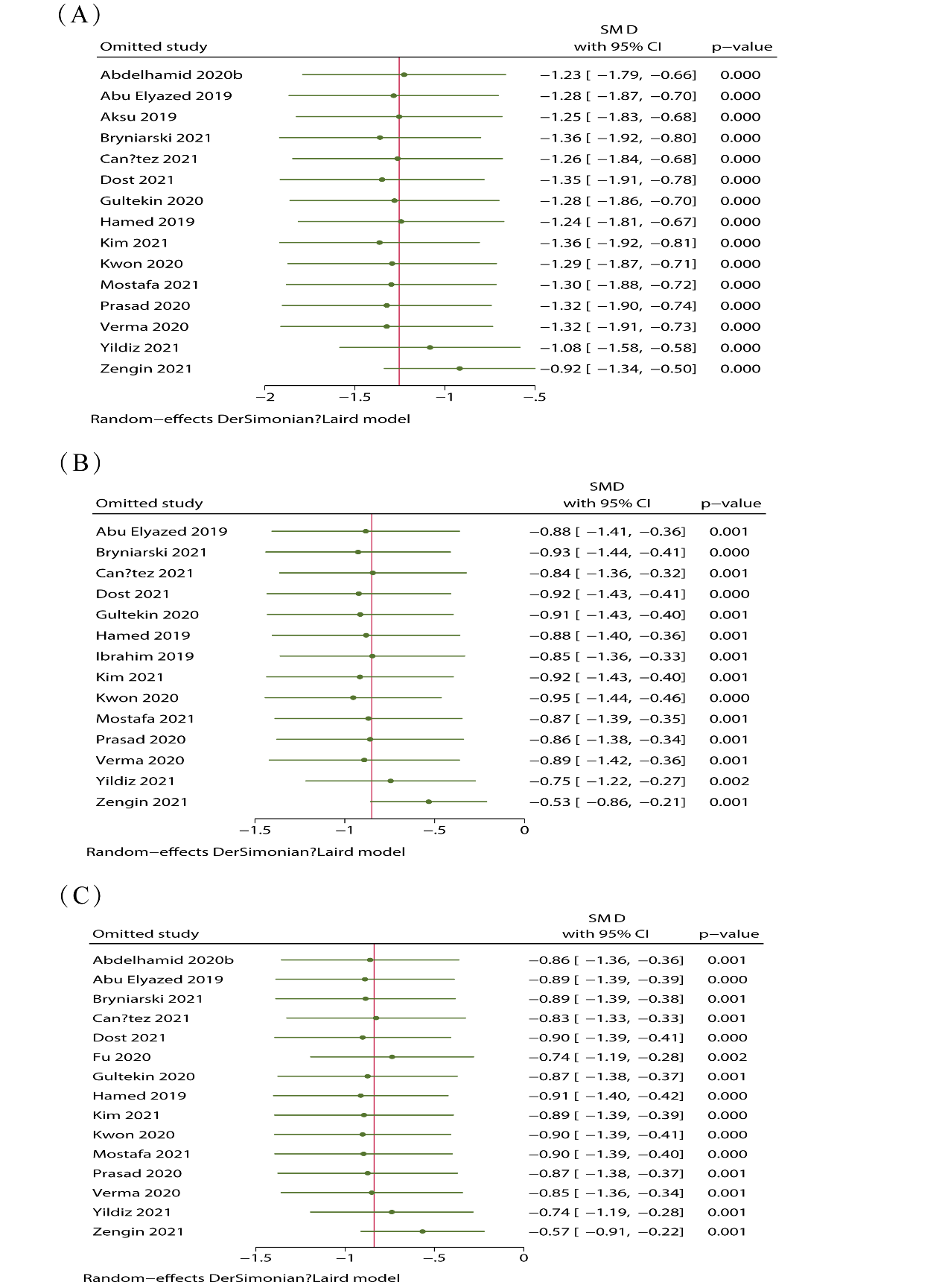


Fig. S1. Sensitivity analyses for pain scores for the ESPB versus placebo in the first 24 h after surgery. (A) Pain score at 6 h after surgery studies. (B) Pain score at 12 h after surgery studies. (C) Pain score at 24 h after surgery studies.


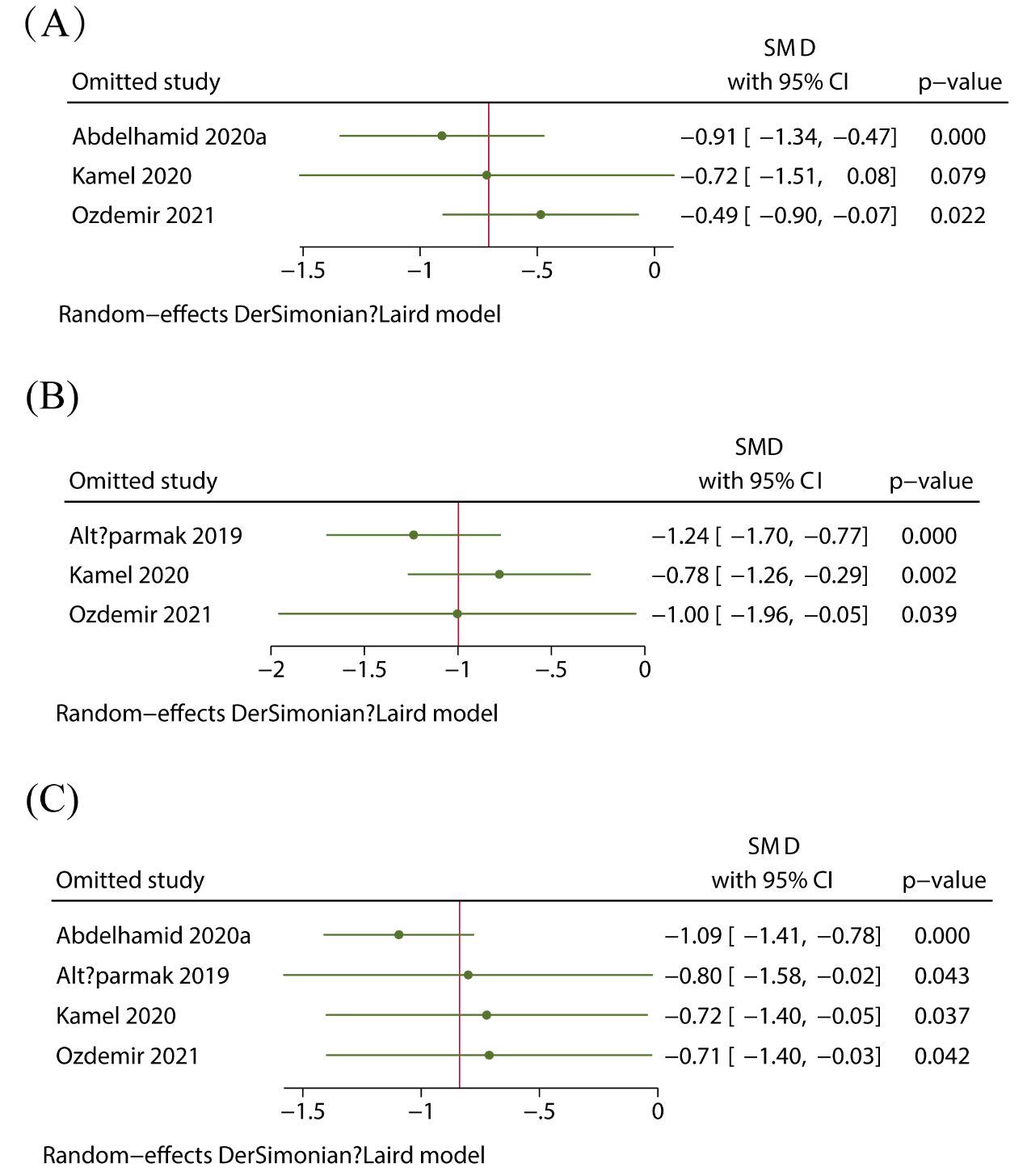


Fig. S2. Sensitivity analyses for pain scores for the ESPB versus TAPB in the first 24 h after surgery. (A) Pain score at 6 h after surgery studies. (B) Pain score at 12 h after surgery studies. (C) Pain score at 24 h after surgery studies.


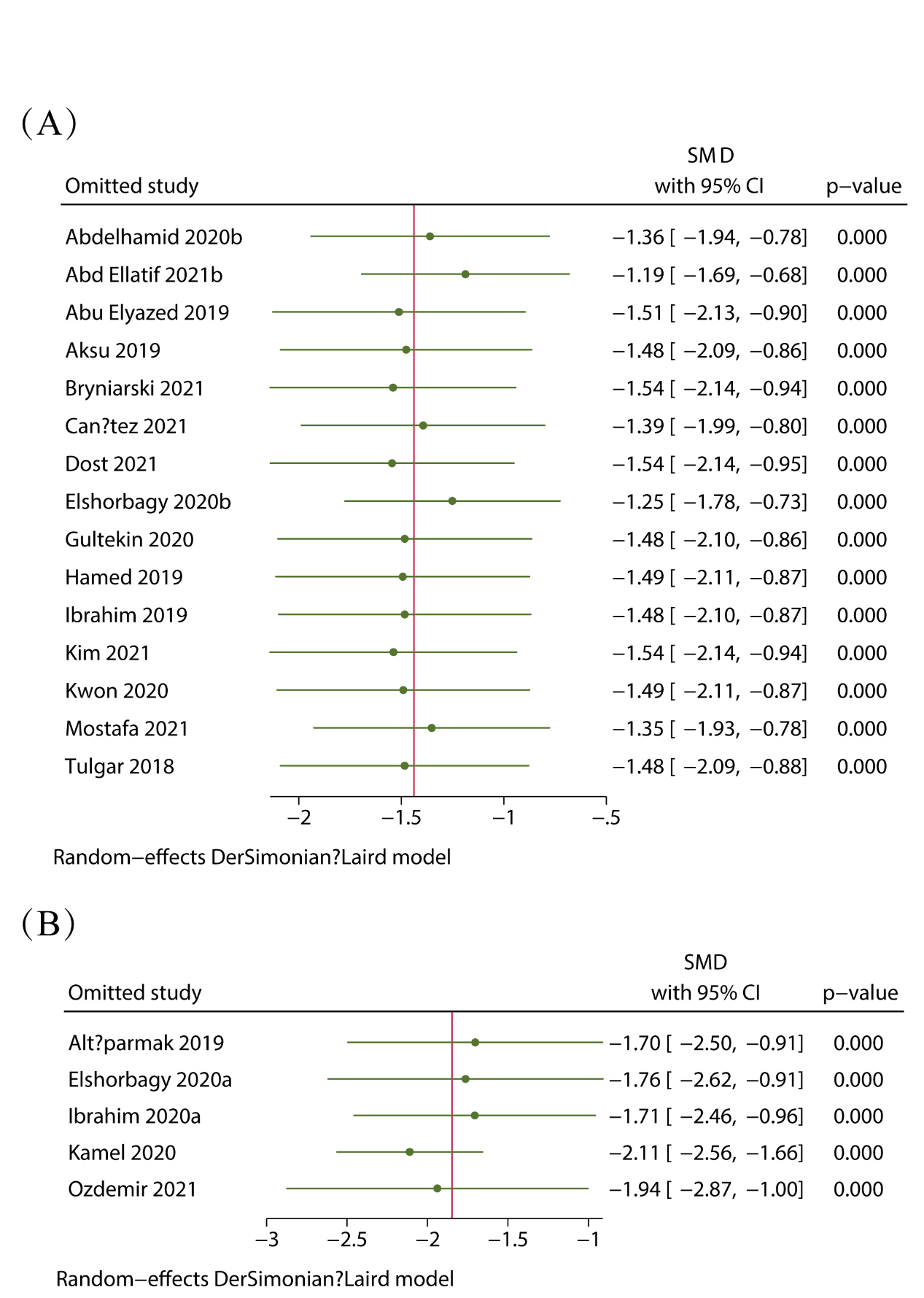


Fig. S3. Sensitivity analyses for the comparison of intravenous morphine equivalents (mg) in the first 24 h after surgery. (A) 24-h cumulative opioid consumption for the ESPB versus placebo studies. (B) 24-h cumulative opioid consumption for the ESPB versus TAPB studies.


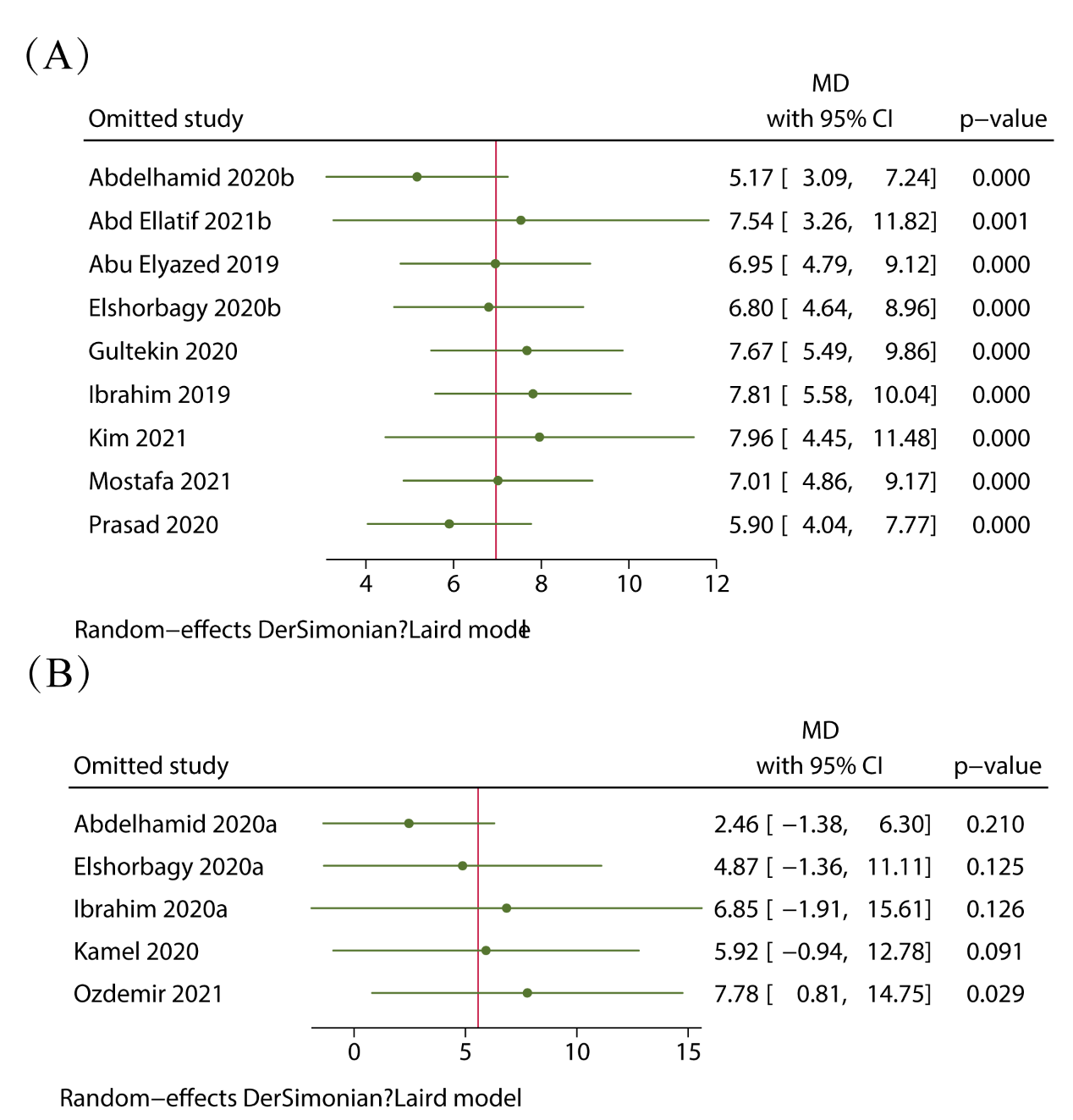


Fig. S4. Sensitivity analyses for the comparison of time to first rescue analgesia (h) after surgery. (A) time to first rescue analgesia for the ESPB versus placebo studies. (B) time to first rescue analgesia for the ESPB versus TAPB studies.


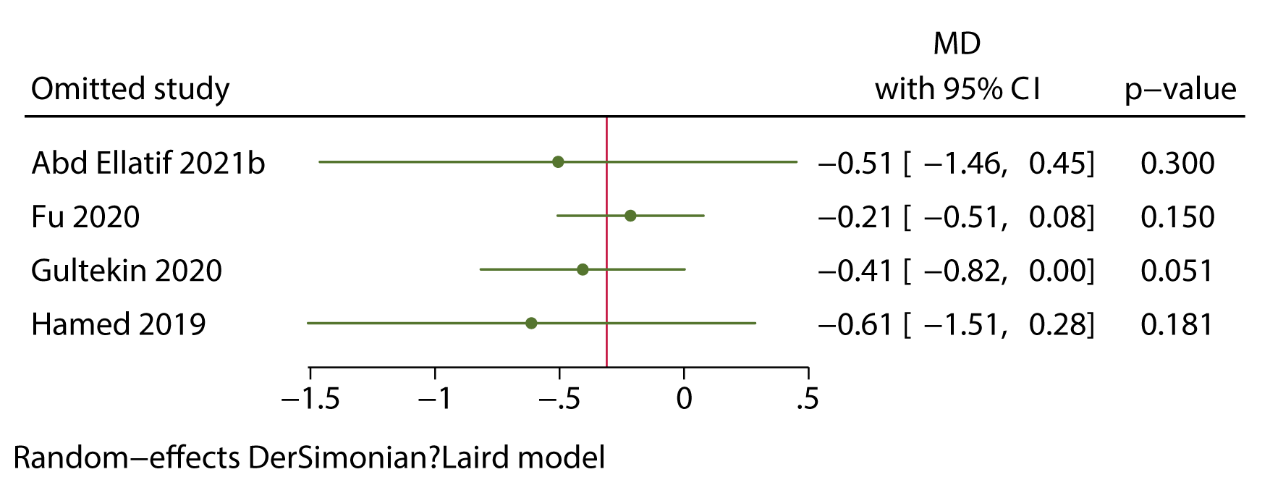


Fig. S5. Sensitivity analyses for length of hospital stay for the ESPB versus placebo after surgery.
